# Epstein-Barr virus-positive, CD30-positive, diffuse large B-cell lymphoma in a patient with angioimmunoblastic T-cell lymphoma

**DOI:** 10.1016/j.jdcr.2022.04.029

**Published:** 2022-05-13

**Authors:** Marc Grossman, Jia Ruan, Cynthia Magro

**Affiliations:** a Department of Dermatology, Yale University, New Haven, Connecticut; b Hofstra/Northwell, Hyde Park, New York, New York; c Department of Internal Medicine, Division of Hematology and Oncology, Weill Cornell Medicine, New York, New York; d Department of Pathology and Laboratory Medicine, Weill Cornell Medicine, New York, New York

**Keywords:** Epstein-Barr virus, large B-cell lymphoma, angioimmunoblastic T-cell lymphoma, AITL, angioimmunoblastic T-cell lymphoma, DLBCL, diffuse large B-cell lymphoma, EBV, Epstein-Barr virus

## Introduction

The development of cutaneous Epstein-Barr virus (EBV)-associated secondary diffuse large B-cell lymphoma (DLBCL) in patients with angioimmunoblastic T-cell lymphoma (AITL) has rarely been described in the literature. We encountered a patient who presented with skin lesions that were diagnosed as being compatible with AITL with subsequent lymph node-based confirmation. Later in his clinical course, he presented with a different skin lymphoma, namely one of diffuse large-cell B-cell lymphoma associated with EBV positivity. The pathogenetic events that lead to the development of B-cell lymphoma in the setting of AITL are discussed; moreover, similar cases of metachronous AITL and cutaneous EBV-positive DLBCL are reviewed.

## Case report

**Fig 1:**
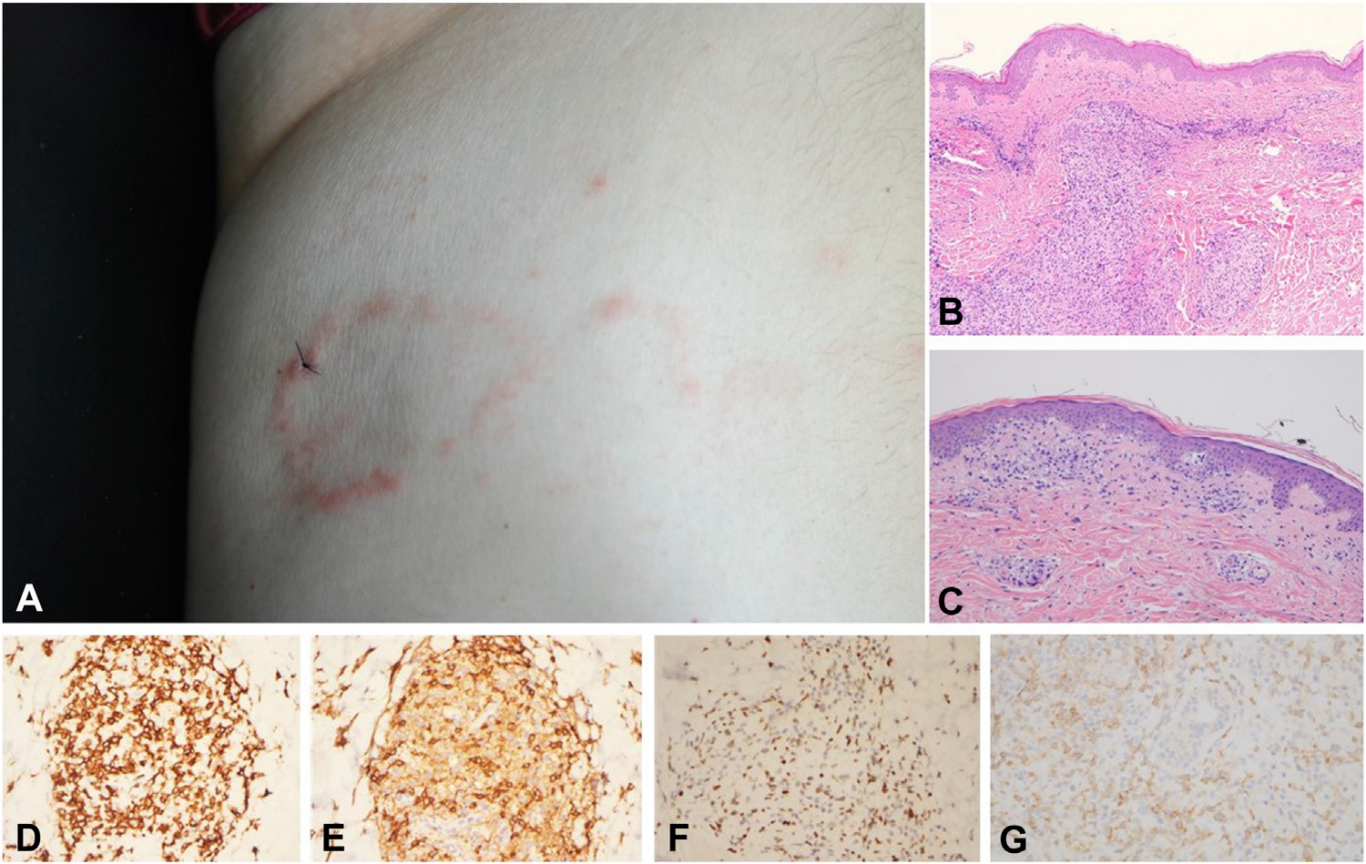
The patient developed an annular plaque on the leg (**A**). A biopsy was obtained and was held to be consistent with angioimmunoblastic T-cell lymphoma (**B-F**). The biopsy exhibited an atypical perivascular lymphocytic infiltrate characterized by pleomorphic small-to-intermediate–sized noncerebriform atypical lymphocytes. Epidermotropism was also noted (**C**). The phenotypic profile was characteristic for angioimmunoblastic T-cell lymphoma, whereby the lymphocytes stained for CD2 (**D**), establishing their T-cell lineage, CD4 (**E**), and exhibited follicular helper T-cell marker-positivity, including BCL6 (**F**) and PD1 (**G**).

A 68-year-old retired teacher had a 6-month history of weight loss and seronegative arthritis of the ankles, knees, wrists, and hands diagnosed as osteoarthritis by a rheumatologist and an orthopedic surgeon, which stopped him from playing golf. Papules, nodules, and annular plaques developed over 2 months on the right shoulder, abdomen, left flank, and left lower eyelid (Fig 1, *A*). He had no lymphadenopathy. He had 18% Monocytosis, whereby his white blood count was 3900 per microliter (normal range, 4000 to 11,000 per microliter of blood). An initial skin biopsy from the upper part of his left arm performed in October of 2018 demonstrated an atypical pandermal and focally epidermotropic intermediate-to-large lymphocytic cell infiltrate (Fig 1, *B* and *C*). The lymphocytes were a heterogeneous mixture of small, intermediate, and larger lymphoid cells, although the large-cell component did not exceed more than 30% of the entire infiltrate. Within the dermis the infiltrate assumed a perieccrine and perivascular disposition. Phenotypic studies showed a predominance of CD4-positive T cells exhibiting a variable loss of CD3, CD5, and CD7 (Fig 1, *D* and *E*); a number of the atypical T cells exhibited positivity for select follicular helper T-cell markers, namely PD1, BCL6, CXCL13, and ICOS (Fig 1, *F* and *G*). The Epstein-Barr virus encoded RNA stain was negative. Less than 5% of the infiltrate expressed CD20. A diagnosis was made of a peripheral T-cell lymphoma with a follicular helper T-cell phenotype likely representing AITL given the accompanying systemic symptoms and mild neutropenia. The T-cell receptor-gamma gene rearrangement and heavy chain immunoglobulin-gene rearrangement studies to identify T and B cell clonality, respectively, were negative. Computerized tomographic imaging demonstrated fluorodeoxyglucose-avid multiple pulmonary nodules, several subcutaneous and intramuscular nodules, and a solitary liver nodule suspicious for lymphoma. Bone marrow biopsy and peripheral blood flow cytometry showed no evidence of lymphoma. One month later he developed an enlarged mass in his right neck that was biopsied and more or less mirrored the malignant T cell process from his right upper arm whereby there was diffuse infiltration of the skin by an atypical T-cell infiltrate that phenotypically mirrored the patient’s left upper arm skin biopsy. T and B cell molecular studies were negative. The sample procured from the head and neck surgeon did not include lymph node tissue. Lung biopsy samples were nondiagnostic because of extensive tissue necrosis precluding morphologic assessment.

**Fig 2:**
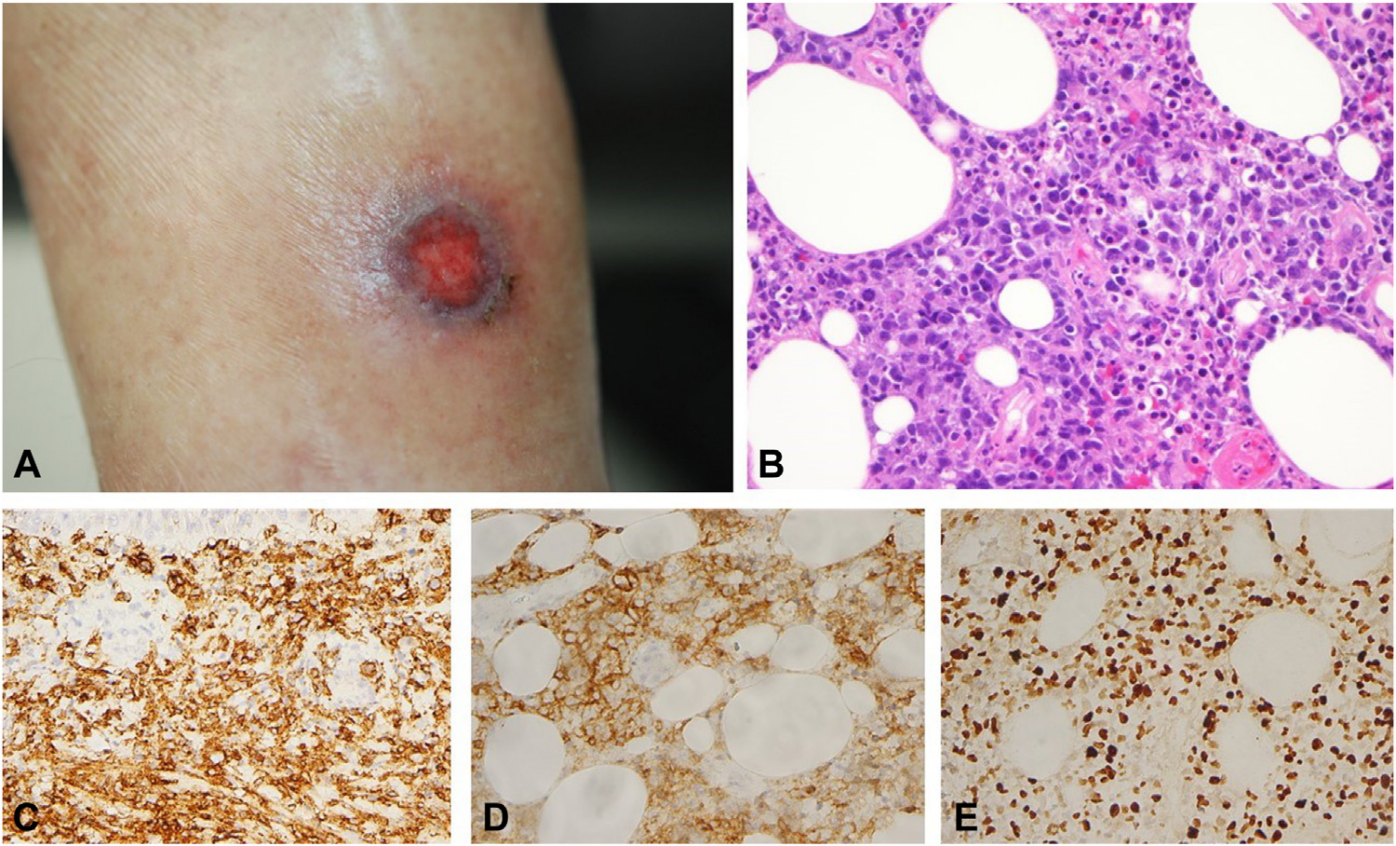
Three weeks after completing chemotherapy, the patient developed a 4-centimeter saucer-like plaque with a 15-millimeter central necrotic eschar on the medial aspect of the left ankle (**A**). A biopsy was compatible with diffuse large B-cell lymphoma as revealed by a diffuse, effacing, dermal and subcutaneous infiltrate of large malignant blastic appearing cells (**B**) highlighted by CD20 indicative of their B-cell ontogeny (**C**), CD30 (**D**), and EBER (**E**).

The patient completed 2 cycles of azacitidine and cyclophosphamide, doxorubicin, vincristine, and prednisone (also known as “CHOP”) and developed demyelination on electromyography. Three weeks after completing his chemotherapy, a 4-centimeter saucer-like plaque with a 15-millimeter central necrotic eschar developed on the medial aspect of the left ankle (Fig 2, *A*). A 4-millimeter punch biopsy from the margin of the ulcer was performed on March of 2019 and demonstrated findings diagnostic of EBV-positive, CD30-positive, lambda light chain-restricted DLBCL 5 months after the original diagnosis of AITL (Fig 2, *B-D*). In particular, the biopsy showed a dermal and subcutaneous infiltrate of large malignant cells with a blastic appearance, whereby the large cells expressed CD20, CD79a, MUM1, and lambda with a number of cells staining positive for CD30; the atypical cells did not express CD10 and BCL6. There was a minor population of small atypical T cells exhibiting a CD4+, PD1+, and BCL6+ phenotype compatible with a residuum of the patient’s known peripheral T-cell lymphoma. A perirenal soft-tissue biopsy showed EBV-positive DLBCL infiltrating adipocytes morphologically and phenotypically identical to the left-ankle tumor. Immunoglobulin Kappa light chain molecular studies were positive, whereas the T-cell receptor-gamma gene rearrangement and heavy chain-gene rearrangement studies were negative. Treatment was switched to cyclophosphamide, doxorubicin, and prednisone (vincristine omitted to minimize neuropathy) plus rituximab for DLBCL. He completed 4 cycles of rituximab, cyclophosphamide, hydroxydaunorubicin hydrochloride (doxorubicin hydrochloride), vincristine (Oncovin) and prednisone plus 2 more infusions of rituximab. End of treatment positron emission tomography/computed tomography scan showed mixed response with resolution of the pelvic mass but new skin nodules. A biopsy of the new skin nodule from his back area demonstrated recurrent AITL. He subsequently initiated treatment with romidepsin and remains in remission from both T-cell and B-cell lymphoma.

## Discussion

AITL represents a post-thymic peripheral T-cell lymphoma often accompanied by multisystem complaints, including polyarthralgias and arthritis, weight loss, fever, polyclonal hypergammaglobulinemia, generalized lymphadenopathy, hepatosplenomegaly, and skin eruptions.^1^ AITL was the first lymphoproliferative disorder recognized to be of follicular helper T-cell derivation.^1^

Follicular helper T cells are a subset of CD4+ helper T cells normally found in the follicular germinal centers of lymph nodes, in which they help promote B-cell expansion and differentiation to long-lived plasma cells and memory B cells. Phenotypically, the follicular helper T cells are characterized by immunoreactivity for programmed death cell protein 1 (PD-1), CD10, B-cell lymphoma 6 (BCL-6), inducible co-stimulator CD278 (ICOS), the receptor for CXCL13 (CXCR5), CXCL13, nuclear staining of nuclear factor of activated T cells (NFAT), and thymocyte selection associated HMG-box (TOX). Only CXCR5 and CXCL13 are truly specific follicular T-cell markers.

AITL is often histologically obscured by a significant degree of B cell infiltration, including small immunoblastic collections of EBV-positive B cells, Reed-Sternberg-like cells, and many plasma cells, including cases where the plasma cells can be light chain-restricted.^2^ Uncommonly, a B-cell lymphoma can emerge. The pathogenetic events that underlie the development of malignant B cell lymphoma in the setting of AITL include EBV infection of transformed B cells coupled with iatrogenic and endogenous immune dysfunction and promotion of B-cell hyperplasia through the neoplastic follicular helper T cell-enriched microenvironment. The commonest type of B-cell lymphoma is the DLBCL, but other forms of B-cell lymphoproliferative disease have been described, including mixed type II cryoglobulinemia and small lymphocytic lymphoma/chronic lymphocytic leukemia.^3^, ^4^, ^5^, ^6^, ^7^, ^8^, ^9^, ^10^ The development of secondary B-cell lymphomas with a DLBCL morphology may portend a more aggressive clinical course with multiple sites of lymphomatous dissemination, including bone marrow, duodenal bulb, lung, ileum, soft tissue, and skin.^3^, ^4^, ^5^, ^6^, ^7^, ^8^

In addition to our case, 5 additional cases have been reported of skin involvement by secondary, EBV-positive, diffuse large B-cell lymphoma in AITL represented by 2 women and 3 men, ranging from 49 to 77 years of age, presenting with a solitary thigh lesion in 1 case and a more diffuse multifocal cutaneous eruption involving the trunk and extremities in the other 4 cases.^3^, ^4^, ^5^, ^6^, ^7^, ^8^ The DLBCL developed 6-21 months after the diagnosis of AITL, including 2 cases in which it developed after completion of AITL chemotherapy. Three patients died of lymphoma, and 2 patients are in remission. One case was lost to follow-up. A summary of these cases is presented in Table I.^3^, ^4^, ^5^, ^6^, ^7^

**Tabel I:** Cutaneous diffuse large B-cell lymphoma in the setting of angioimmunoblastic T-cell lymphoma

Number	Age(y)/sex of patient	Clinical presentation of AITL	Interval from presentation of AITL to DLBCL	Clinical presentation of DLBCL	F/U	Reference
1	65 Man	Inguinal LAD Maculopapular rash Hepatosplenomegaly No biopsy -EVA -Hypergammaglobulinemia	19 mo	Irregular plaques and nodules, 0.5-2.5 c, Trunk sepsis Skin Bx+	Lost to F/U	Yang et al (2012)^5^
2	77 Man	Cough Cervical LAD Bx+ Inguinal erythematous papules and plaques, scattered trunk	8 mo	Shooting neuropathy centrally Necrotic plaque on thigh Bx+	Died 3 mo after dx DLBCL	Lee et al (2016)^4^
3	49 Woman	Enlarged left conical node Bx+ Hepatosplenomegaly No skin lesions	21 mo	Plaques/nodules with ulcers trunk and extremities Bx atypical lymphoid proliferation 2 mo later C/7 nodule	Died 5 mo after dx DLBCL	Chen et al (2018)^6^
4	47 Man	Macular/papular lesions Diffuse LAD Splenomegaly Inguinal LN Bx+	After 2 cycles of CHOP 14	Painful ulcerated abscesses and nodules, bilateral axillae, Bx+	Remission at 9 mo	Poon et al (2019)^7^
5	68 Woman	B symptoms Hepatosplenomegaly LAD Bx+ Eosinophilia No skin lesions	7 mo	Multiple pruritic erythematous plaques, arms/back Bx+	Died 14 mo after dx AITL	Simsek et al (2019)^3^
6	68 Man	Seronegative arthritis Papules, nodules Monocytosis Bx+	6 mo	4-cm saucer-like plaque, centrally necrotic Bx+	In remission	Present study

*AITL,* Angioimmunoblastic T-cell lymphoma; *BX,* biopsy; *DLBCL,* diffuse large B-cell lymphoma; *dx,* diagnosis; *LAD,* lymphadenopathy; *LN*, lymph node; *F/U,* follow-up; *CHOP,* cyclophosphamide, hydroxydaunorubicin, oncovin, prednisone.

+, positive.

In summary, the B cell-promoting microenvironment in the setting of AITL leads to significant B-cell hyperplasia, ranging from a state of reactive B-cell hyperplasia to one of frank B-cell neoplasia. The skin defines a potential initial site of presentation of both AITL and secondary B-cell lymphoma developing in AITL. This case emphasizes the importance of rebiopsying patients with AITL, when there is a change in their existing cutaneous disease and/or the development of new lesions.

## Conflicts of interest

None disclosed.
